# Short-Term Outcome of Isolated Kidney Transplantation in Children with Autosomal Recessive Polycystic Kidney Disease: A Case Series and Literature Review

**DOI:** 10.3390/clinpract14010003

**Published:** 2023-12-21

**Authors:** Ratna Acharya, Kiran Upadhyay

**Affiliations:** 1 Department of Pediatrics, Nemours Children’s Hospital, Orlando, FL 32827, USA; ratna.acharya@nemours.org; 2 Division of Pediatric Nephrology, Department of Pediatrics, University of Florida, Gainesville, FL 32610, USA

**Keywords:** autosomal recessive polycystic kidney disease, isolated, kidney transplantation, children

## Abstract

Autosomal recessive polycystic kidney disease (ARPKD) is often associated with hepatobiliary disease in the form of hepatic fibrosis and/or Caroli disease. Combined liver–kidney transplantation (CLKT) is a transplant modality of choice in children with both end-stage renal disease (ESRD) and severe hepatic disease. However, there is no consensus on whether children with ARPKD-associated ESRD without severe hepatic disease can be treated with isolated kidney transplantation (KT) without the need for CLKT. We retrospectively studied the efficacy of isolated KT in children with ARPKD without severe hepatic disease, and followed the course of hepatic disease post KT. This is a single-center study of three children with ARPKD and ESRD who underwent isolated KT. None of them had severe hepatic disease at the time of KT. All children were clinically diagnosed with ARPKD in the immediate postnatal period. All had hepatic fibrosis of varying degrees and two had intrahepatic biliary duct (IHBD) dilatation. None had gastrointestinal (GI) bleed, portal hypertension or cholangitis. Two children had preemptive KT. Pre-transplant unilateral or bilateral native nephrectomy were performed for two children, and one underwent unilateral native nephrectomy at the time of KT. The median creatinine clearance at a median post-KT follow-up of 24 months was 60.3 mL/min/1.73 m^2^. The two-year graft and patient survival were both 100%. Post KT, all three patients continued to demonstrate evidence of hepatic fibrosis and IHBD on sonogram; however, none of them were either evaluated for or required liver transplantation given normal synthetic liver function and absence of portal hypertension or other severe hepatobiliary disease. There were no adverse events observed such as cholangitis, GI bleed, or multiorgan failure. Hence, an excellent short-term graft and patient survival was demonstrated in this study of children with ARPKD and mild to moderate hepatic disease who received isolated KT. Long-term follow-up and larger studies are important to assess the efficacy of isolated KT in this subset of children with ARPKD.

## 1. Introduction

Autosomal recessive polycystic kidney disease (ARPKD) is caused by mutations in the *PKHD1* gene that encodes a ciliary protein called fibrocystin [1]. In ARPKD, association with hepatobiliary disease is frequently seen in the form of congenital hepatic fibrosis and/or intrahepatic biliary duct dilatation (IHBD) [2,3]. Caroli disease is defined as a pure cystic, saccular, fusiform, segmental, and non-obstructive dilatation of large IHBD. Caroli syndrome is the combination of Caroli disease along with congenital hepatic fibrosis and/or cirrhosis, portal hypertension, or esophageal varices. Severe hepatobiliary disease manifests as recurrent episodes of cholangitis, decompensated cirrhosis, upper gastrointestinal (GI) bleed, refractory portal hypertension and/or hepatopulmonary syndrome [4].

In children with ARPKD who progress to end-stage renal disease (ESRD), it is important to carefully assess the severity of hepatic disease to determine whether a concomitant liver transplantation along with kidney transplantation (KT) is also beneficial. Studies have reported that if there is an isolated ESRD without portal hypertension or severe hepatic disease, isolated KT may be sufficient [5]. In those without evidence of ESRD but with severe hepatobiliary disease, isolated liver transplantation is an option [4]. In children with both ESRD and severe hepatic disease, combined liver kidney transplantation (CLKT) may be an important treatment modality, providing immunoprotective effect to the kidney offered by the donated liver [6,7]. Sequential liver kidney transplantation is another option but with an added risk of higher rates of rejection compared to CLKT. However, data regarding the timeline of development of post-KT severe hepatobiliary disease requiring liver transplantation are lacking in the cohort of children with ARPKD who undergo isolated KT initially.

We studied three children with ARPKD with hepatic fibrosis (*n* = 3) and mild IHBD (*n* = 2) who underwent isolated KT. We described the two-year outcome on patient and graft survival and also studied the post-KT course of hepatobiliary disease in these children.

## 2. Methods

This was a single-center case series of three children with ARPKD and ESRD who underwent isolated KT.

## 3. Case Series

Three children underwent deceased donor KT at the median age of 6 years 7 months (Table 1). All had ARPKD diagnosis during the neonatal/infancy period based upon sonogram and clinical manifestations. Two underwent genetic tests confirming ARPKD. Patient 1 had *PKHD1* gene variants (c.9719G > A and c.3766del in each of the copies of the gene, heterozygous, pathogenic) and Patient 3 had the *PKHD1* gene variant (c.107C > T in both copies of the gene, heterozygous, pathogenic). One child required ventilatory support during the neonatal period for respiratory distress due to pulmonary hypoplasia and enlarged kidneys. Two were females. One child started renal replacement therapy with peritoneal dialysis at the age of 17 months. Following an episode of fungal peritonitis, the modality was changed to hemodialysis a year later. One child received enteral nutrition via a gastrostomy tube. None of them had upper GI tract bleeding, episodes of cholangitis or cholelithiasis. Physical examination showed no overt ascites or hepatosplenomegaly. Synthetic liver function remained normal in all three children. Hepatic doppler sonogram showed echogenic, heterogenous liver parenchyma with hepatopetal flow in all children and mild IHBD in two children (Table 2). Elastography showed varying degrees of hepatic fibrosis. None of them underwent liver biopsy pre-KT.

Two children had preemptive KT (estimated glomerular filtration rate (eGFR) at the time of KT: 10–15 mL/min/1.73 m^2^). Two out of three children had pre-transplant unilateral nephrectomy that they underwent between the age of 6 weeks and 15 months for respiratory compromise and refractory hypertension, and another native nephrectomy at the time of KT. The third patient underwent unilateral native nephrectomy at the time of KT. The histology of the kidney in all cases showed that the renal parenchyma was diffusely distorted by innumerable varying-sized thin-walled cysts and absence of solid components within any of the cystic lesions. One patient underwent liver biopsy at the time of KT which showed diffuse ductal plate malformations consisting of bile duct structures with somewhat irregular contours and foci of mild dilatation. The portal tracts/fibrous septa were without significant inflammation, interface activity or edema. There was no lobular inflammation or steatosis. A trichrome stain showed portal expansion by dense fibrosis including broad fibrous septae.

The induction immunosuppression (IS) consisted of Thymoglobulin^R^ (*n* = 2) and basiliximab (*n* = 1). There was an immediate graft function. Maintenance IS consisted of tacrolimus and mycophenolate mofetil with a steroid withdrawal protocol. Two patients were started on prednisone later due to presence of donor specific antibodies (DSA) which was detected during surveillance DSA monitoring as per the Center’s protocol. This occurred while lowering the IS for BK viremia. In these two children, the BK virus DNA PCR peaked at 1080–1520 copies/mL (normal: <390 copies/mL, blood, ARUP laboratories, Salt Lake City, UT, USA). However, the allograft function remained stable at that time. The DSAs became undetected after several weeks. 

## 4. Outcome

All patients had functioning allografts during a median transplant vintage of 24 months (21–27 months) with a current median eGFR of 60.3 (59.8–109.3 mL/min/1.73 m^2^, CKiD U25 eGFR). The median height percentile at the time of KT was 3 (0.01–20, median z score: −1.83 (−4.06 to −0.82)) which increased to a median of 24 (0.01–82, median z score: −0.71 (−4.28 to 0.94)) without growth hormone therapy during the most recent median follow-up period of 24 months. Blood pressures remained stable in all except one patient who continued to require antihypertensive therapy.

In the post-KT period, hepatobiliary disease remained stable in all three children. There were no episodes of cholangitis or cholelithiasis, upper GI bleed, hematemesis, melena, hepatopulmonary syndrome or hepatic encephalopathy. Physical examination continued to show an absence of overt ascites or hepatosplenomegaly (although two children had sonographic evidence of mild hepatosplenomegaly). Liver enzymes and serum bilirubin remained within normal range. Blood counts showed an absence of leukopenia or thrombocytopenia. Doppler liver sonogram continued to show evidence of mild hepatomegaly, echogenic heterogenous parenchyma, mild IHBD in two children, and hepatopetal flow. Shear wave elastography continued to show persistent mild to severe hepatic fibrosis during follow-up. Given stable liver function, none of the three children required liver transplantation.

## 5. Discussion

In ARPKD, about 50% of children progress to ESRD during the first decade of life [6]. Hepatic fibrosis is seen early in life. Chronic liver disease (CLD) presents as portal hypertension, splenomegaly and variceal hemorrhage [6]. The onset of CLD is variable and difficult to predict as opposed to ESRD. Available studies have shown that the average age of portal hypertension in children is about 8.3 years [8]. Some of the treatment options are isolated KT with careful monitoring of the liver function in those with relatively mild hepatic disease, or KT followed by sequential liver transplantation or CLKT in those with both ESRD and severe hepatic disease. Chandar et al. described an optimal approach of transplantation in patients with ARPKD with or without severe hepatic disease [8].

### 5.1. Isolated Kidney Transplantation

The children presented in this report had preserved synthetic liver function and only mild hepatobiliary disease at the time of KT. The isolated KT significantly shortens both the intra-operative and post-operative care time as opposed to CLKT and may be considered in those with stable hepatic function. This is supported by other studies which have shown that most children who received KT for ARPKD-associated ESRD have stable synthetic liver function and no evidence of severe hepatic disease at the time of KT, and hence isolated KT may be sufficient [9]. In contrast, Beunoyer et al. reported the clinical course of neonatal ARPKD in 10 children and observed that early-onset severe hepatic disease was common [10]. In their cohort, 60% had portal hypertension and Caroli disease, and 30% had cholangitis. A total of 80% were transplanted (isolated KT) prior to the age of 3 years. Despite the evidence of severe hepatic disease in a significant portion of the cohort, all children underwent isolated KT. In the post-KT period, 20% required surgical intervention for hepatobiliary disease. One child required liver transplantation 3.7 years after KT. The patient and death-censored graft survival were 89% and 100%, respectively, at a mean of 6.1+/−4.5-year post isolated KT. Some other reports also have suggested that 40% of patients with ARPKD have both severe kidney and liver disease at the time of KT [2]. Hence, there seems to be a variable onset of presentation of hepatic disease in ARPKD patients [3]. Also, there does not seem to be a correlation between severity of kidney disease and liver disease [2]. In addition, the time interval between the diagnosis of liver disease and requirement of liver transplantation in children with ARPKD is unclear. In one study, among the seven children with fibrocystic liver disease, three had associated PKD but without advanced chronic kidney disease and hence there was no indication for KT [4]. Among these three children with both fibrocystic liver disease and PKD, the median patient age at diagnosis of fibrocystic liver disease was three years (range: 5 months–8 years). The median age at liver transplantation was ten years (range: 9 years–12 years). Hence, the median timeline from diagnosis of liver disease to liver transplantation seems to be around seven years. However, very few studies seem to have looked at the timeline from the diagnosis of hepatic disease to requiring liver transplantation among the cohorts of ARPKD patients who initially underwent isolated KT. Davis et al. analyzed the pediatric kidney transplant registry data and showed that two children with ARPKD with mild hepatic disease who underwent isolated KT first (at age 9.4 years and 5.3 years respectively) underwent a liver transplantation at 15.1 years and 5.10 years but both died within 2 months of liver transplantation [9]. Hence, the median timeline for liver transplantation from the time of isolated KT seems to be around 3.3 years. In our study, all three children had stable liver function without evidence of clinical severe hepatic disease in the short duration of 2 years post isolated KT and hence did not require liver transplantation.

In those who undergo isolated KT, the graft and patient survival are comparable between patients with non-ARPKD and ARPKD-related ESRD [9]. However, sepsis associated with hepatobiliary disease, accentuated by post-KT immunosuppression, is the most common etiology of mortality after isolated KT in ARPKD [5,9]. In a single-center study, Khan et al. looked at morbidity from congenital hepatic fibrosis after isolated KT in 14 children (mean age 8.3 years) with ARPKD during a mean follow-up of 14.5 years [5]. One- and five-year patient survival was 93% and 86%, respectively. One- and five-year graft survival was 87% and 70%, respectively. In these children, the progression of hepatic disease through childhood occurred in 79% of cases, and included hypersplenism, esophageal varices with GI bleed and progressive IHBD dilatation. The mortality from progressive hepatic disease occurred in 29% of cases and accounted for 80% of the deaths. The causes of death were hepatic failure immediately post KT, septicemia related to bile duct dilatation, and multiorgan failure. One patient had CLKT and two were re-transplanted with kidney. Hence, patients who undergo isolated KT need close monitoring for worsening of portal hypertension and progression of IHBD dilatation.

### 5.2. Isolated Liver Transplantation

The overt clinical hepatocellular dysfunction in ARPKD may not be typically seen until it is too late despite sonogram evidence of hepatobiliary disease [8]. The clinical manifestations of hepatobiliary disease are seen in 46–85% of children including hepatosplenomegaly, hepatic fibrosis, echogenic and heterogenous liver, portal hypertension, esophageal varices, protein losing enteropathy, and recurrent ascending cholangitis [2,3]. The most common indications of liver transplantation in children with ARPKD are recurrent episodes of cholangitis which can occur in association with Caroli disease or syndrome, decompensated cirrhosis, refractory portal hypertension and hepatopulmonary syndrome [4,11].

Ko JS et al. studied seven children with congenital hepatic fibrosis, Caroli disease, and Caroli syndrome (three children had ARPKD) who underwent liver transplantation [4]. The indications of transplantation were recurrent cholangitis, decompensated cirrhosis, and refractory portal hypertension. Both the liver graft and patient survival rates were 100% at a median follow-up period of two years. In three children with ARPKD, the mean serum creatinine levels increased from 0.53 mg/dL at the time of transplantation to 0.91 mg/dL during the last follow-up.

Chapal et al. presented the results and outcome of kidney and/or liver transplantation in a series of 14 children and young adults with ARPKD [11]. Eleven patients had isolated KT and three had liver transplantation (kidney first followed by liver in two and CLKT in one, 7–20 years after isolated KT). Recurrent and/or severe cholangitis was the indication for liver transplantation. The authors concluded that pre-emptive liver transplantation may be a therapeutic option in ARPKD patients with severe hepatic disease.

### 5.3. Combined Liver–Kidney Transplantation

The common indications of CLKT in children are ARPKD, primary hyperoxaluria type 1 (PH1), methylmalonic acidemia, atypical hemolytic uremic syndrome, α1-antitrypsin deficiency, glycogen storage disease type 1a, tyrosinemia, and severe hepatorenal syndrome [12,13,14]. CLKT accounts for 1–2% of all pediatric liver transplantation, with one third of recipients being less than 5 years old and two thirds being 6–17 years old [13].

In CLKT from the same donor, the liver allograft has been shown to offer immunologic protection to the kidney allograft leading to lower incidence of rejection [7,15]. A few suggested hypotheses are that the liver absorbs the lymphocytotoxic antibodies, and that the circulating alloantibodies are neutralized by the soluble class I HLA-G antigens produced by the transplanted liver [7]. The five-year graft survival rate has been reported to be as high as 80–100% in CLKT [16,17].

Brinkert et al. reported data on eight children with ARPKD who had CLKT [6]. The median age at the time of CLKT was 10.1 years and the median follow-up was 4.6 years. All children had clinical signs of portal hypertension and abnormal sonogram findings with presence of hepatic fibrosis. The patient survival rate was 100% and graft survival was 72% and 88%, respectively, for liver and kidney. This is in concurrent with other studies which have shown that the long-term graft survival in CLKT is similar, although the patient survival is not superior compared to isolated KT or liver transplantation [18]. One study compared the renal function recovery in children undergoing CLKT for PH1 vs. ARPKD and reported that those with PH1 receiving CLKT had delayed recovery of renal function compared with those children with ARPKD, possibly due to mobilization of systemic oxalate [19]. Hence, the post-transplant renal recovery seems to be acceptable in CLKT for ARPKD.

One of the major drawbacks of CLKT is the surgical challenge of the donor–recipient size mismatch, especially in small children. Perera et al. compared the renal recovery between children who weighed less than 15 kg vs. the ones who weighed more than 15 kg and who underwent CLKT [20]. The children in both groups (<15 kg and >15 kg) demonstrated improved renal function at 12 months after CLKT. They concluded that CLKT in small children resulted in comparable outcomes in comparison to the relatively larger-sized children [20].

The limitations of this study include a small number of cases and a short duration of follow-up. Longer follow-up studies are needed to assess the timeline of development of severe hepatic disease requiring liver transplantation in children with ARPKD who initially undergo isolated KT. In our study, hepatic disease remained stable and none of the children required liver transplantation in the short two-year follow-up period.

## 6. Conclusions

Isolated KT may have a role in the condition of children without severe hepatic disease such as evidence of portal hypertension or recurrent cholangitis. Longer-term studies are needed to look at the post-KT onset of severe hepatic manifestations in this cohort of children. Also, larger comparative studies are needed to examine the efficacy of isolated KT versus CLKT in those with ARPKD-related ESRD and less severe hepatic disease.

## Figures and Tables

**Table 1:** PD: Peritoneal dialysis; KT: Kidney transplantation.

	Age at Transplant	Nephrectomy Status	Dialysis Status	Hypertension	Post-Transplant Follow-Up Period
Patient 1	4 year 7 months	Single native nephrectomy at 15 months, another native nephrectomy at the time of KT	PD started at 17 months of age	No antihypertensive medication prior to KT	21 months
Patient 2	8 years 4 months	No pre-transplant nephrectomy, unilateral native nephrectomy at the time of KT	Pre-emptive	No antihypertensive medication prior to KT	27 months
Patient 3	6 years 8 months	Single native nephrectomy at 6 weeks, another native nephrectomy at the time of KT	Pre-emptive	Labetalol	24 months

**Table 2:** Hepatic manifestations at the time of transplant. ALT: Alanine aminotransferase; AST: Aspartate aminotransferase; IHBD: Intrahepatic biliary duct; EHBD: Extrahepatic biliary duct; METAVIR: Meta-analysis of histological data in viral hepatitis.

	Doppler Sonogram	Shear Wave Elastography Sonogram	Liver Function
Patient 1	Mild hepatomegaly, heterogenous echogenic liver parenchyma with minimal IHBD dilatation; patent portal vein with hepatopetal flow, normal splenic size	Mild to moderate liver fibrosis with pediatric METAVIR score of F2–F3	AST 39 IU/L (0–37 IU/L), ALT 23 IU/L (0–35 IU/L)
Patient 2	Heterogenous coarse echotexture of the hepatic parenchyma, mild IHBD dilatation, borderline EHBD dilatation, patent main portal vein with hepatopetal flow, normal splenic size	Mild to moderate liver fibrosis with pediatric METAVIR score of F2–F3	AST 23 IU/L (0–37 IU/L), ALT 16 IU/L (0–35 IU/L)
Patient 3	Heterogenous echotexture of liver, patent main left and right portal vein with hepatopetal flow, no IHBD, spleen mildly enlarged	Moderate to severe liver fibrosis with pediatric METAVIR score of F3–F4	AST 24 IU/L (0–37 IU/L), ALT 13 IU/L (0–35 IU/L), GGT 19 U/L (5–16 U/L))

## Data Availability

Data is contained within the article.
